# Dead or alive: sediment DNA archives as tools for tracking aquatic evolution and adaptation

**DOI:** 10.1038/s42003-020-0899-z

**Published:** 2020-04-07

**Authors:** Marianne Ellegaard, Martha R. J. Clokie, Till Czypionka, Dagmar Frisch, Anna Godhe, Anke Kremp, Andrey Letarov, Terry J. McGenity, Sofia Ribeiro, N. John Anderson

**Affiliations:** 1 Department of Plant and Environmental Sciences, University of Copenhagen, Thorvaldsensvej 40, DK-1871 Frederiksberg C, Denmark; 2 Department of Genetics and Genome Biology, University of Leicester, University Road, Leicester, LE1 7RH UK; 3 Ecology, Evolution and Biodiversity Conservation, KU-Leuven, Charles Deberiotstraat 32 - box 2439, 3000 Leuven, Belgium; 4 University of Birmingham, School of Biosciences, Birmingham, B15 2TT UK; 5 Department of Marine Sciences, University of Gothenburg, Box 461, SE 405 30 Göteborg, Sweden; 6 Finnish Environment Institute (SYKE), Marine Research Centre, P.O.Box 140, 00251 Helsinki, Finland; 7 Winogradsky Institute of Microbiology RAS, prospect 60-letiya Oktyabrya 7/2, 117312 Moscow, Russia; 8 School of Life Sciences, University of Essex, Wivenhoe Park, Colchester, CO4 3SQ UK; 9 Glaciology and Climate Department, Geological Survey of Denmark and Greenland (GEUS), Øster Voldgade 10, 1350 KBH-K København, Denmark; 10 Department of Geography, Loughborough University, Loughborough, Leicestershire LE11 3TU UK; 11 Present Address: University College Copenhagen, Humletorvet 3, 1799 Copenhagen, Denmark; 12 Present Address: Leibniz Institute for Baltic Sea Research Warnemünde, Department of Biological Oceanography, Seestraße 15, 18119 Rostock, Germany

**Keywords:** Ecosystem ecology, Palaeontology, Applied microbiology

## Abstract

DNA can be preserved in marine and freshwater sediments both in bulk sediment and in intact, viable resting stages. Here, we assess the potential for combined use of ancient, environmental, DNA and timeseries of resurrected long-term dormant organisms, to reconstruct trophic interactions and evolutionary adaptation to changing environments. These new methods, coupled with independent evidence of biotic and abiotic forcing factors, can provide a holistic view of past ecosystems beyond that offered by standard palaeoecology, help us assess implications of ecological and molecular change for contemporary ecosystem functioning and services, and improve our ability to predict adaptation to environmental stress.

## Abstract (web summary)

Ellegaard et al. discuss the potential for using ancient environmental DNA (eDNA), combined with resurrection ecology, to analyse trophic interactions and evolutionary adaptation to changing environments. Their Review suggests that these techniques will improve our ability to predict genetic and phenotypic adaptation to environmental stress.

## Introduction

**Fig. 1:**
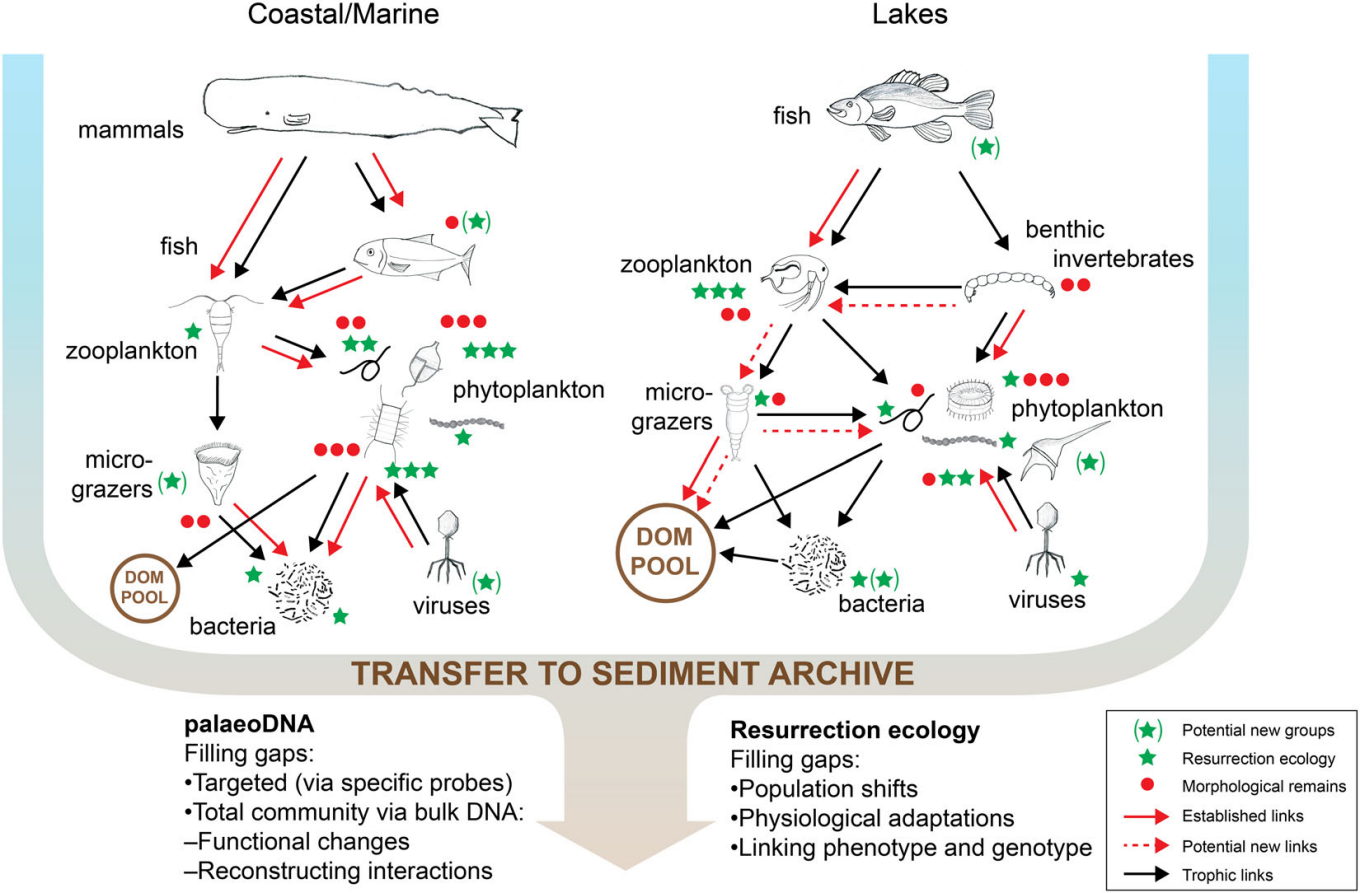
A schematic food-web in a limnic and coastal marine system. This figure shows where we have data on resurrection ecology series and indicates the food-web and interaction gaps in in the palaeoecological record, which sed-eDNA has the potential to fill in, to reconstruct food-webs, possibly through association networks, as suggested by^16^ for lake ecosystems. Extracting, respectively, DNA and live propagules from dated sediment core has the potential to greatly enhance both the amount and types of information that we can gain about evolutionary and adaptive processes, by filling different information gaps in the paleo-ecological record. Green stars indicate the organism types for which genetic and phenotypic time-series have already been established from resurrected resting stages; the more stars, the larger the existing dataset. A green star in parenthesis indicates that viable resting stages have been recorded from old sediment layers, but no timeseries data published. Red circles indicate organism types for which there is information based on morphological remains (“traditional” palaeoecology); the more circles, the larger the existing dataset.

**Fig. 2:**
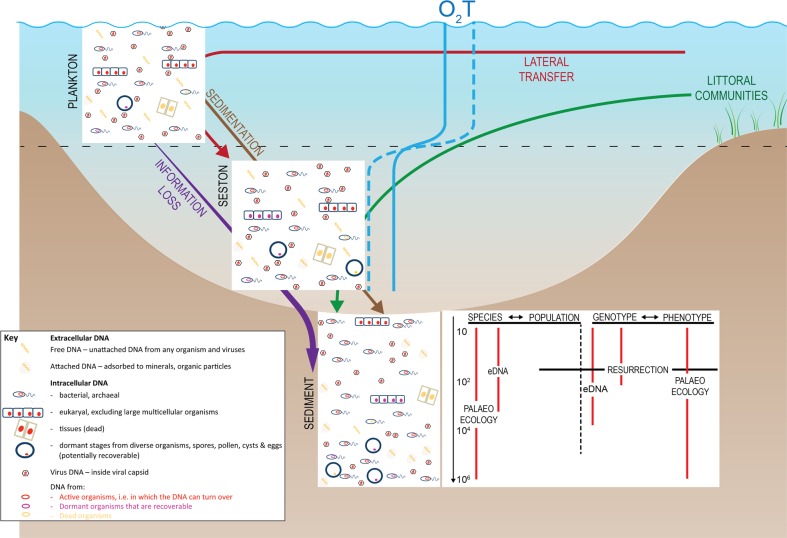
Schematic illustration of change in relative abundance of DNA due to taphonomic processes. This figure illustrates the processes affecting DNA distribution, degradation and/or preferential preservation during the transitions from the pelagic to the benthic zones, and from the surface sediment to the deeper sediment. The approximate timescales of preservation of different fractions of the sediment record is also illustrated.

Undisturbed lake and marine sediments are natural archives of past changes in biota and their environment, and when dated, they offer the opportunity of reconstructing past changes in e.g. both primary and secondary production and community composition^1,2^. Analysing organismal remains in freshwater and marine sediment cores provides a long-term perspective of ecological change and has a long history in both pure science and applied contexts^3^ (Table 1). In the more traditional approaches to the palaeoecology of aquatic systems, microfossil analysis is accompanied by a number of geochemical proxies including lipid biomarkers, pigments and isotope composition (Table 1). The interpretation of these archives, i.e. the science of palaeoecology, is dependent on understanding contemporary ecological controls as well as the sedimentation environment and its context. The remains of a diverse range of organisms, from viruses to mammals, can be preserved in lake and marine sediments (Fig. 1). However, the degree to which they faithfully reflect changing abundance and community composition varies enormously depending on taxon preservation capacity^4^, the depositional environment, including sedimentation rate, and distance to the depositional site (Fig. 2).

**Table 1:** A summary of the strength and weaknesses of the different approaches to reconstructing aquatic ecosystems and pheno- and genotypic variability over time in aquatic systems.

Method	Strengths	Weaknesses	References
Palaeoecology based on fossil organisms	• >10^7^ year time scales	• No genotype information	^93,94^
	• Large datasets are available	• Labour intensive	
	• Potentially quantitative	• Only some groups/species preserved	
Geochemical (bio)markers	• >10^7^ year time scales	• No genotype information	^95^
	• Large databases available	• Potential for porewater mobility	
	• High throughput	• Lacks the taxonomic specificity of DNA sequences	
	• Potentially quantitative		
Sedimentary eDNA timeseries	• So far, ~10^5^ year time scales	• No direct phenotype information	See references in text
	• Cover all domains of life	• So far, above population level	
	• High throughput	• Few reference sequences	
	• Sequence data has the potential to link specifically to taxa or traits	• Potential for porewater mobility	
	• Potentially quantitative (qPCR; so far only ~100 years)	• New bacterial and archaeal signals overprint the paleo-sequences, due to in situ growth	
		• Risk of chimeras & contamination	
		• Risk of bias in extraction	
Resurrection ecology	• So far, 10^1^-10^2^ time scales (much longer for Bacteria and Archaea)	• Labour intensive	See references in text
	• Linking genotype and phenotype directly	• Only some species preserved	
	• Applicable at population level	• Potential bias in survivability, but single-cell approaches possible	
	• Potentially quantitative

In the past decade, the fields of resurrection ecology and environmental DNA (eDNA; Box 1) have developed to a degree which now enables us to complement these traditional analyses with analyses of temporal change at the genetic and genomic levels. This has the potential to enhance our understanding of evolutionary processes in aquatic systems and organisms. We can now evaluate impacts of environmental stressors on both genotypic and phenotypic responses of individual species, as well as on interactions between species and on whole communities. These new approaches can thereby expand our capacity for predictive modelling to project future change, and for impact assessments.

**Fig. 3:**
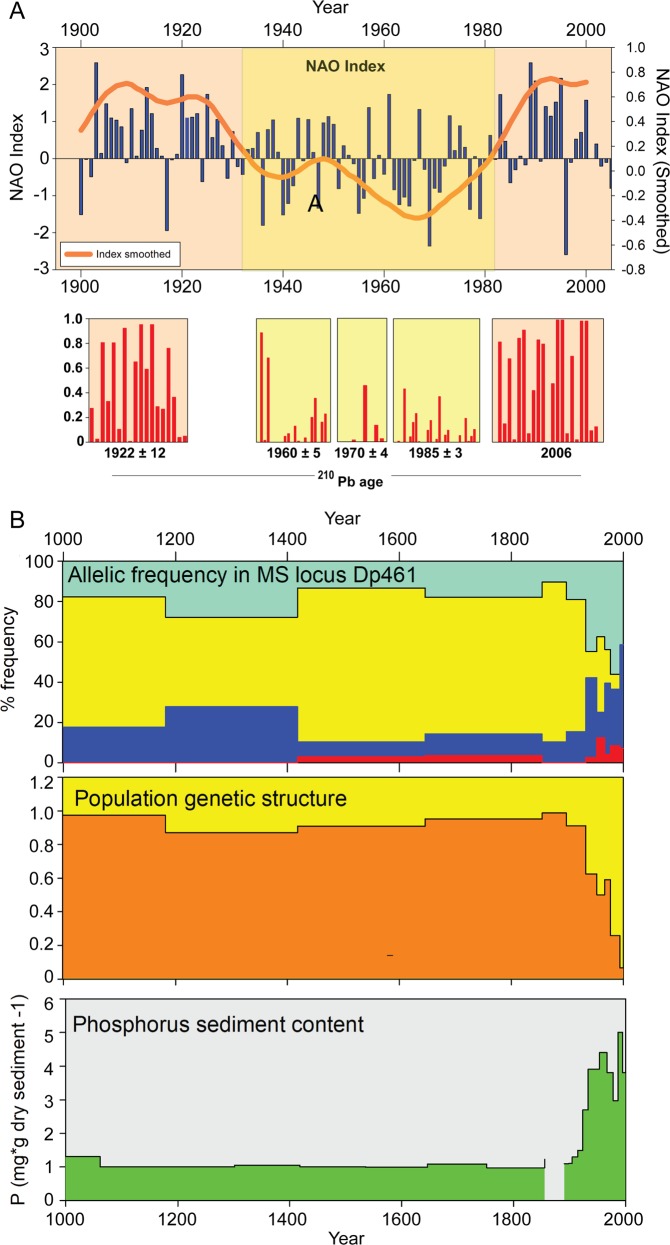
Resurrection ecology can be used to generate time series of population genetic data to test hypotheses of adaptation to temporal stressors. The same strains, from which the DNA was extracted, can be used for side-by-side, or common-garden, tests of phenotypic/physiological response to the same stressors. Here we show modified versions of figures from Lundholm et al.^29^ (**a**) and Frisch et al.^5^ (**b**), both showing population structure plots. **a** Analysis of population genetic response of the phytoplankton Pentapharsodinium dalei in a Swedish fjord to environmental change associated with changes in the index of the North Atlantic Oscillation (NAO), which affects, among other things, salinity and water-column stability. **b** Analysis of population genetic response of the herbivore Daphnia pulicaria in a lake to changing phosphorus concentrations through time.

Resurrection ecology is the study of temporal series of revived resting stages from dated sediment layers. Many planktic (as well as benthic) species form dormant or resting stages (propagules), which accumulate over time in aquatic sediments. Such sediment propagule banks contain eggs, spores, cysts and other resilient structures from all domains of life (Bacteria, Archaea and Eukarya) as well as viruses. Importantly, resting stages from many of these taxa can remain viable for centuries^5,6^ (Fig. 3). From terrestrial environments, a similar phenomenon is seen in seeds, but in most terrestrial environments, time series and reliable chronologies are difficult to achieve, as soil disturbance, due to bioturbation for example, will obscure the age of different depths and oxygenation will enhance organic matter mineralisation. However, a few studies have been able to follow depth series in specialised environmental settings such as marginal water bodies (e.g., in solifluction lobe; ca. 150 years max. age)^7^ and cedar glades^8^. Nonetheless, continuous, temporally structured, well-preserved archives covering multiple trophic levels and with strict age-control are unique to aquatic sites with undisturbed sedimentation. Temporal genetic signals of change can also be analysed by extracting total sedimentary DNA (Box 1). Similar to other biomarkers, DNA may be archived in aquatic sediments, but as described below (see Fig. 2), the degree to which this occurs depends heavily on deposition and preservation conditions. Thus, sediment-archived DNA may be either extracellular, in dead tissue/cells, or inside living organisms (within dormant propagules or active microbes). Each of these sources of DNA can be used for detecting genetic change that reflects responses to environmental change in natural populations and communities at a range of trophic levels.

Box 1: Terminology Resurrection ecology is a rapidly evolving field of research and consists of reviving long-term resting stages from sediment archives and using these to create time-series of culture strains, that can be used to quantify both genetic and phenotypic response (a recent special issue in the journal *Evolutionary Applications* (vol. 1, 11, 2018) is dedicated to this field).

Resting (or dormant) stages are produced during the life cycle of a range of plankton species, as well as sediment- and soil-dwelling Bacteria, Archaea and and heterotrophic protists, and include spores, cysts, eggs and other poorly described structures found in Bacteria and Archaea. They are characterised by resistant walls, and a reduced metabolism, arresting further development. A mandatory state of resting is termed dormancy, or, in zooplankton, diapause, whereas a reversible state (dependent on external stimuli) is termed quiescence (see e.g. Radzikowski^36^).

Resurrection is the experimentally induced reversion of buried resting stages from their inactive state of reduced metabolism to an active state of growth and reproduction. In Bacteria, Archaea, Fungi and phytoplankton the process is termed germination, where active cells emerge or directly grow from resting stages (e.g. akinetes of cyanobacteria, spores of bacteria and diatoms or cysts of dinoflagellates and chrysophytes). Zooplankton can hatch from dormant eggs, resuming growth and reproduction. Germination and hatching of historic resting stages are typically triggered by a change in environmental conditions including oxygen, light and temperature (see e.g. Ellegaard and Ribeiro^12^).

Aquatic propagule banks are natural reservoirs of viable plankton resting stages deposited in bottom sediments. Such banks have a large significance for community and population dynamics as well as for the evolutionary resilience of species to rapid environmental fluctuations.

DNA in aquatic sediments may originate from both dead and living organisms, and may be either allochthonous (transported) or autochthonous (produced in situ). In this paper, we focus only on DNA produced by aquatic organisms (autochthonous). DNA in aquatic sediments may fall within several loosely defined terms, including environmental (eDNA), sedimentary (sedDNA), ancient (aDNA) and bulk DNA, and may include either extracellular and intracellular DNA, or a combination of both. In most cases, eDNA extracted from bulk sediment will be a combination of genetic material in metabolically active cells and released from dead cells, while others refer to eDNA as the genetic material obtained directly from the environment (including sediments), without any obvious sign of biological source material^96^. These definitions do not specifically account for DNA preserved in sediments inside structurally intact cells with varying degrees of metabolic activity, such as those of phytoplankton and zooplankton dormant stages. Structurally intact cysts, spores and eggs in aquatic sediments may be either dead or physiologically active, and still preserve valuable DNA material. If DNA is extracted from bulk sediments, it will have a mixed origin from both inside (possibly living and dead cells) and outside cells.

DNA obtained from resting stages (either directly or after resurrection) has been referred to as aDNA by some authors. aDNA is a term typically applied to DNA recovered from ancient remains of dead organisms (often bones, hair, etc. but also ancient environmental samples). Others define ancient DNA on the basis of the quality of the DNA rather than the age^97^, meaning that historical DNA with no signs of fragmentation or degradation is not aDNA by this definition. The application of this term is therefore controversial when referring to DNA obtained directly from viable resting stages, or from resurrected strains, but may be correct when referring to DNA recovered from non-viable resting stages. As the demarcation between dormancy and death is not easily assessed in resting stages, this terminology deserves careful consideration. Here, we use the term sedimentary-environmental DNA (sed-eDNA) for bulk DNA extracted from dated sediment layers.

## Combining approaches, and moving from single species to interactions

So far, almost all studies based on resurrection ecology have targeted single species (see the compilation in a special issue of Evolutionary Applications vol. 1, 11, 2018). Here, we present the potential for moving this field toward the more complex level of species interactions by documenting how we can achieve resurrection time-series for new groups of organisms (to fill gaps at different levels of the food-web; Fig. 1).

Recent reviews^9^ have covered the state-of-the-art on both environmental and ancient DNA, and the body of work on resurrection ecology of single species (see summary below) was presented in a recent special issue of Evolutionary Applications (vol. 1, 11, 2018). Therefore, here we focus on how to develop the real, and mainly untapped, potential benefits of combining the two approaches. We show how the two lines of evidence from resurrection ecology and temporal series of sedimentary eDNA complement each other, and how analysing both in the same sediment record can give synergistic effects and facilitate much deeper insights into past evolutionary and adaptive trajectories and interactions.

Finally, we show how insights from more traditional palaeoecology, including preservation and spatio-temporal variability in deposition signals, can help in interpreting the genetic signals from sediment cores. Building on these insights allows the evaluation of the robustness of different chronologies and the validity of the emerging signals—to what extent do they represent real change and to what extent are they affected by preservation and taphonomic processes?

eDNA analyses can greatly expand the taxonomic coverage of traditional palaeoecological reconstructions, and of resurrection series. It can also be used to specifically target signals from resurrection model-species and to expand the range of coverage temporally. Resurrection ecology, on the other hand, adds the dimension of phenotypic and physiological response to the genetic information, and enables tracking of adaptive trait shifts. The two types of data thus offer information on different fractions of past communities.

We argue that judicious application of these emerging methods in resurrection ecology and sedimentary eDNA make it possible to track impacts of environmental change on aquatic ecosystems at centennial, and even millennial, time scales, and to link temporal phenotypic with genotypic responses, thus enabling us to assess adaptibility, resilience and evolvability across whole ecosystems. We highlight the opportunities that are resulting from the rapid development of sophisticated methodology but also discuss the current limitations that need to be addressed in order to achieve the full potential of temporal reconstruction of genetic and phenotypic responses to environmental change.

## Resurrection ecology: studies on temporal series of intact cells/propagules

Dormant stages of many species can remain alive in aquatic sediments for decades and even centuries^10,11,12^ (see examples in Fig. 3), and it is possible to “resurrect” past populations from these resting stages buried in undisturbed sediment via hatching of zooplankton eggs or germination of phytoplankton cysts and spores (Box 1), and potentially spores and other dormant forms of Bacteria, Archaea, Fungi, as well as, potentially, other unicellular heterotrophic organisms. Resurrection ecology, which is the science based on testing temporal, revived, series of strains, now encompasses several trophic levels of the aquatic food web (Figs. 1 and 3) and can vastly increase our understanding of responses to changes at both phenotypic and genotypic levels. In addition, DNA can be obtained from resurrected strains in a quantity and quality that make in-depth analysis of full genome sequences feasible, providing enormous potential for insights into evolutionary genomics^13^. Genomic information from living propagules will be a key resource for reconstructing marine and freshwater biological community responses to environmental change, e.g. by discovery of loci of adaptation by applying genome-wide association studies. Resurrection studies further allow reconstruction of past phenotypes in the laboratory to directly study historical populations, thereby assessing trait changes over time as well as their transcriptomic basis^14^. Resurrection ecological studies thus occupy a unique niche by documenting actual processes of century-scale adaptation at both genetic (Fig. 3) and phenotypic levels. So far, almost all studies based on resurrection ecology have targeted single species and, to date, there has been a preference for studying large, identifiable, long-lived resting stages such as *Daphnia* eggs and dinoflagellate cysts. Below, we document the potential for achieving resurrection time-series for new groups of organisms, to fill gaps in food-chain levels, and discuss the potential for expanding resurrection ecology to include interspecific and trophic-level interactions. We thus argue that reviving resting stages from multiple organism groups and trophic levels from the same site can now make it possible to reconstruct interactions and co-adaptation trajectories over evolutionarily relevant time scales (Fig. 1).

### Viruses—potential for resurrection series

Viruses are a ubiquitous component of the biosphere associated with other life forms in all ecosystems. Lakes and their sediment have abundant viral communities which are are dominated by bacteriophages^15^ and the sediment documents the past microbial populations from the over-lying water body. Importantly, valuable information on virus biology contained within this sediment archive can be revealed from the direct resurrection of the viruses, for example by isolating phages that infected the cyanobacterium *Microcystis* up to 50 years ago. Recently, Baltic Sea sediments were found to harbour diverse, allochthonous and autochthonously produced viruses down to the deepest point investigated of 37 m, representing about 6000 years since deposition^16^. The extensive literature documenting phage particles’ morphology from deep-sediment layers suggests that the phages are intact^16^, and so if the correct host strains are available, these viruses could be cultured. Past populations of viruses and their hosts could thus be resurrected and used to infer co-evolutionary dynamics over time.

### Bacteria, Archaea and heterotrophic protists

Longevity of some microbes over millions of years is now widely recognised^17–19^. Resurrection of Bacteria and Archaea is thus not new but differs from studies on larger, morphologically conspicuous organisms in that, usually, enrichment culture targetting a range of taxa is used rather than first separating individual organisms. Even though bacterial palaeoecological resurrection studies started in 1990, using (allochthonous) bacterial spores as palaeo-indicators of agricultural land use^20^, and resting stages (akinetes) of cyanobacteria have been germinated from old sediment layers^21,22^, the full potential of this strategy has yet to be realised. Wunderlin et al.^23^ confirmed the value of spore-formers (using non-resurrection approaches) in that their abundance relative to total bacteria increases with sediment age. Still, our understanding of the potential for DNA preservation in, as well as survival of, resting stages of different bacterial taxa, is inadequate^6^, and the plethora of techniques to improve microbial cultivation and selectively enrich for specific functional groups has barely been used in palaeoecology. Heterotrophic protists, such as flagellates, cilates and amoebae, may also be present in sediments, both in encysted and actively metabolising states. A few studies have cultivated amoebae found downcore in aquatic sediments^24^, and other studies have shown that ciliates may survive tens of thousands of years in permafrost soils^25^. However, to the best of our knowledge, no genetic and/or phenotypic studies of these organisms have been carried out on sediment time-series.

### Phytoplankton

Resting stages are found in many species and in most groups of phytoplankton^12^, but so far resurrection ecological studies on time series have been restricted to a few, marine, species: the dinoflagellates *Alexandrium* spp., *Pentapharsodinium dalei* (example in Fig. 3), and *Apocalathium malmogiense*^26^ and the diatom *Skeletonema marinoi*. These have been used to explore the impact of environmental conditions (e.g. salinity^6,27,28^ and eutrophication^29^) on population genetic dynamics over multidecadal time scales, as well as phenotypic adaptation to changed environmental conditions (e.g.^6,26,27^). These examples illustrate that it is possible to trace evolutionary change of genotypes as well as phenotypic reaction norms of different traits in response to environmental state using monoclonal cultures established from revived resting stages and kept in the laboratory side-by-side with modern strains. These phenotypic responses can then be linked to the corresponding genomic data.

### Metazoa, mainly zooplankton

The most comprehensive dataset of resurrection ecology is probably that from crustaceans with dormant stages. The bulk of these studies have been carried out on the water flea *Daphnia*, e.g. to identify species invasions^30,31^ and to track effects of eutrophication^32,33^ (example in Fig. 3). Many other invertebrates are present in propagule banks but have rarely been exploited for genetic or resurrection studies^34^. However, Epp et al.^35^ were able to relate changes in different genotypes of *Brachionus* rotifers to dramatic environmental change such as water level or the deposition of volcanic ashes over 100 years. Using dormant copepod eggs retrieved from lake sediment, Makino et al.^36^ recovered 21 haplotypes using 28S ribosomal DNA. The brine shrimp *Artemia* is also being explored as a model for resurrection ecology in higher salinity environments^37^.

Resurrection ecological studies on *Daphnia* spp. have shown how a population evolved (and subsequently lost) its resistance to toxic cyanobacterial blooms over a few decades^38^, documented a historical change in the phenotypic plasticity of phosphorus physiology in response to anthropogenic eutrophication^5^ and showed changes in phototactic behaviour^39^ and other traits^40^ in response to changes in predation pressure. The comparison of transcriptomic responses of resurrected 10- and 700-year old *Daphnia* isolates allowed identification of gene networks and key functional drivers involved in the evolutionary adaptation to eutrophication^14^. Recently, the first attempts to perform whole-genome amplification of DNA from dormant stages have been made^41^—a method that potentially facilitates whole-genome sequencing even of propagules that cannot be hatched or germinated.

For fish, aDNA has been extracted from remains of otoliths^42^ or scales^43^ from museum archives, but, although these remains can accumulate in the sediment, we are not aware of the use of sediment-buried fish remains to reconstruct historic evolutionary adaptation to an environmental change at the genetic or genomic level.

### Next steps

Potential pitfalls in the study of resurrected time series regarding e.g. issues of the non-representative nature of revived populations^44–46^ and differential survival can be addressed in the planning phase of a study. Thus, biases and artefacts related to specific phenotypes can be detected by the analysis of multiple sediment cores^39,47^, cores from greater depths or from anoxic sediments not exposed to early cues for germination/hatching. In the case of phyto- and zooplankton resting stages, rapidly developing single-cell sequencing approaches may help to identify germination and survival biases^48^, and the application of beneficial bacteria and their (or other) extracts may enhance germination^49^. Issues of adaptation to culture conditions^50,51^ can be circumvented by phenotyping the cultures soon after germination.

Currently the main datasets for zooplankton originate from lakes while the main datasets for phytoplankton derive from marine coastal embayments. However, as mentioned above and illustrated in Fig. 1, this bias can be overcome by intensifying the effort to germinate multiple species from different trophic levels from the same site, allowing the evolutionary dynamics of biotic interactions to be studied if both organisms deposit viable resting stages in the same sediment. One of the few studies to adopt this approach revealed co-evolutionary dynamics between *D. magna* and its microparasites by resurrecting host and parasite populations from different time periods^47^. Similarly, the influence of viral infections on the changing abundance of cyanobacteria such as *Microcystis* in eutrophic lakes could be shown by linking pigment concentration with estimates of cyanophage infection (sensu Hargreaves). The multi-faceted aspect of eutrophication on freshwater foodwebs and species interactions has been addressed by coupling resurrected *Daphnia* with bacterial infections (*Pasteuria ramosa*) against a background of changing food quality associated with increased nutrient load. Reyserhove et al.^52^ show that genetic differentiation in *Daphnia* is affected by food availability, and ultimately influences parasite virulence. Finally, an example from the marine realm: as described earlier, resting stages of the dinoflagellate *Pentapharsodinium dalei* can survive ca. 100 years in undisturbed sediment cores in temparate and arctic regions^27^. *Parvilucifera* is a genus of well-known parasites that are strain-specific for *P. dalei*^53^. Germinating infected strains of the dinoflagellate from separated time-slices, isolating the parasite, and attempting to re-infect strains with parasites of a different age can give novel insights into co-evolution in real systems at multidecadal to century time scales.

Going further, adopting a comparative genomics approach will improve our understanding of adaptation to environmental change by targeting specific genes or genomic regions across taxa present in the sediment archive and linking this to specific phenotypic responses.

## DNA sedimentary archives and their taphonomy

The potential of using eDNA sediment time series in ecology was recently covered in a thorough review by Balint et al.^9^. Therefore, here we focus specifically on two points: (1) the potential for linking eDNA data with resurrection ecology, and (2) methological issues that need more attention to tap the full potential for reconstructing planktic communities and interactions.

### The taphonomy of DNA

As mentioned in the Introduction, valid interpretations of environmental signals derived from aquatic sediment timeseries rely heavily on an understanding of sedimentological processes (e.g. depositional environment and rates) and process of preservation and degradation (taphonomy). These considerations, perhaps even more critically, apply to the analysis of DNA archived in aquatic sediments. Thus, a thorough understanding of the factors influencing DNA preservation must underpin the field of research into sediment timeseries of eDNA.

The probability of enzymatic and abiotic degradation of DNA increases with time, hence the importance of rapid burial. This particularly applies to extracellular DNA, but even intracellular DNA will be damaged if the cell has ceased active repair. In the dark and anoxic conditions typical of benthic sediments, hydrolysis (depurination and deamination) is likely to be the main abiotic process contributing to DNA decay^54^. Anoxia^55^, minimal bioturbation^55^ and low temperatures^56^ are conducive to an excellent sedimentary DNA archive. Extreme salinity also has an impact on DNA preservation, as shown for brines from deep-sea anoxic hypersaline lakes^57^, especially those rich in chaotropic salts which can be effectively sterile and therefore excellent for preserving biomolecules^58^. Furthermore, hardwater lakes provide good preservation due to calcite formation, which supports rapid sedimentation^59^.

A large fraction of sediment DNA is extracellular, e.g. 90% in deep-sea sediments^60^ and 31% in lake sediments^61^. In fact, extracellular DNA in aquatic sediments is the largest global reservoir of DNA, with implications for ecosystem functioning^60^ as an important source of carbon, nitrogen and especially phosphorus. Many microbes assimilate these elements from DNA^62^ and in some cases depolymerised DNA is used as a source of energy^62,63^.

Extracellular DNA has differential bioavailability, depending on whether it is free or adsorbed to sediment particles. DNA bound to minerals or organic matter can constitute the majority of extracellular DNA (e.g. >95% in marine sediments^64^), and attachment can enhance its preservation. Cation bridging of DNA to clay minerals is a major mechanism by which DNA bioavailability is reduced, due to the large surface area to volume ratio of clay minerals and, for some clay minerals, their laminar structure, whereby DNA can be adsorbed between clay layers^54,65^. In addition to reducing bioavailability of DNA, nucleases adsorb to minerals^66^, potentially reducing their activity^54^. Extreme conditions may facilitate preservation of cells/DNA over hundreds, thousands or even millions of years, as found with evaporite minerals, such as halite^17^. There is still a lot to learn about how mineral type and organic matter composition, coupled with other factors such as ionic composition, influence the early diagenetic processes of extracellular sedimentary DNA^54,65^.

### Temporal extent of DNA archives in aquatic sediments

These preservation issues non-withstanding, DNA signals of a large variety of organism groups have been traced continuously over millenial time scales in aquatic sediment archives. Most studies have extracted bulk DNA from the sediment without distinguishing between DNA inside live cells/resting stages and extracellular DNA. Our focus is on the potential for reconstructing planktic aquatic ecosystems and we refer to other studies for catchment-derived DNA signals (of e.g. trees and other vegetation) stored in lake sediments^67^.

With regard to eukaryotic aquatic organisms, temporal changes in sed-eDNA have been documented over time scales of thousands to tens of thousands of years. For lakes this was reviewed by Domaizon et al.^59^. In a marine setting, Lejerzerowicz and co-workers^68^ recovered DNA from deep-sea sediment cores collected in the South Atlantic, dated to about 32.500 years ago, including DNA from taxa that do not fossilise well and undetermined taxa. A more recent study used sed-eDNA to estimate the colonisation date for white fish in a Swedish lake^69^. eDNA approaches therefore offer the potential to assess the impact of environmental change across taxonomic groups, over long temporal scales, and potentially with a taxonomic resolution unavailable by traditional microfossil approaches. However, many authors have also highlighted limitations^70^ (see also Fig. 2) and questioned the reliability of DNA archives from sediments as a stand-alone proxy^71^. Instead, most researchers advocate a combination of DNA evidence and palaeoecological approaches as the way forward (i.e. using DNA as one proxy within a multiproxy study)^70,72,73^. Indeed, as indicated in the previous section, the mechanisms of DNA preservation are sometimes unclear and even counterintuitive. This point is illustrated by a sediment core study^74^ from the stratified Watts Basin in Antarctica, which reported a 10-fold decline in diatom DNA and a 10,000-fold decline in dinoflagellate DNA over 2700 years. However, there was no ecological explanation for this difference, and quantification of the dinoflagellate biomarker, dinosterol, did not support this massive decline. Therefore, these findings were attributed to preferential preservation of diatom DNA within resting stages, which were also found in the sediment record, together with potentially greater lability of dinoflagellate DNA due to their lack of histones^74^. Applying parallel studies of the two types of temporal genetic signals derived from eDNA and resurrection ecology can further illuminate these issues.

### Bacteria, Archaea, Fungi and Viruses

Bacteria, Archaea and to a lesser extent Fungi, pose both an opportunity and a threat to the field of sed-eDNA. The opportunity is that, as for other organisms, their sed-eDNA can be used for temporal reconstruction of communities, with at least two requirements: firstly, that the environment is favourable to preservation (Fig. 2); secondly, that the DNA is from a group that would be present and functional in the water column but not the sediment. The threat comes from the ability of many microorganisms to function in sediments, with two primary, interconnected effects: (1) they increase the bioavailability and degradation of organic matter, including DNA and other biomarkers derived from supposedly archived organisms; (2) they multiply in the sediment, and thus alter the microbial community composition, i.e. mixing up the modern (autochthonous) and ancient sed-eDNA. With increasing depth and time, most DNA derives from autochthonous microbes that are adapted to the present deep-sediment conditions in both marine^75^ and lacustrine^76^ environments. However, exceptional preservation may occur as discussed earlier, for example in the case of 217,000-year-old DNA derived from phototrophic *Chromatiaceae*^77^ and 2700-year-old DNA from green-sulfur bacteria (*Chlorobium*)^74^. Naturally, most bacterial sed-eDNA studies have focussed on phototrophs that are allochthonous to the deep sediment, including cyanobacteria^78,79^, *Chlorobi*^74^ and *Chromatiaceae*^80^.

Phylogenetic genes are valuable when there is a near-unambiguous taxonomy-trait relationship, e.g. as found in cyanobacteria, where photosynthesis is a group trait, notwithstanding the capacity for other modes of energy generation in some cyanobacterial lineages such as those detected in the deep subsurface^81^ and per-alpine lakes^82^. More recently, genes encoding enzymes with a specific function are being used, such as a cyanobacterial gene (mycA) coding for the synthesis of the toxin microcystin, which has been used to identify where potentially toxic cyanobacterial blooms had occurred in perialpine lakes^78^. Similarly, the particulate methane monooxygenase gene (*pmoA*) in anoxic lake sediments has been used to infer past aerobic methane oxidation in the water column^83^. DNA extracted from *Bacillus* spores in a lake sediment archive has shown a rise in abundance of antibiotic resistance genes from the 1960s for tetracycline resistance and 1970s for sulfonamide resistance, demonstrating the value of such studies in understanding the historical legacy of antibiotic use^84^.

Analysis of sed-eDNA has the potential to provide a unique window into host-parasite evolution, such as that between cyanobacterial *Planktothrix* chemotype hosts and their parasitic chytrid fungi^85^. The lake sediment record showed stable co-existence of host and parasite, raising questions about spatial avoidance (infection refuges) or evolution of resistance chemotypes in *Planktothrix*^85^. The diversity and population dynamics of sediment viruses could also be assessed using metagenomic, culture-independent methods by probing specific groups. Many bacterial taxa are associated with particular phage groups, so their diversity can be explored using primers specific to conserved genes such as the capsid protein or portal protein genes that are currently used as a tool to assess marine cyanophage diversity^86^. The phage-encoded genes for bacterial metabolism proteins may also provide clues to deciphering past ecological conditions. For example, some cyanobacterial phages harbour the genes for phosphorus uptake proteins^87^. A third way to explore past viral dynamics is possibly the newly developed method that enables virus sequences to be linked to bacteria using the frequency and abundance of CRISPR sequences in both the viruses and bacteria^88^.

### Next steps

Increasing phylogenetic coverage (including reference sequences) and amount of sequence data and implementing links to phenotypic change by targeting functional genes will help us towards the goal of temporal reconstruction of entire ecosystems and their response to change,

Applying multiproxy approches, and building on insights from palaeoecological studies can give an increased understanding of preservational issues and sampling techniques, leading to more robust and reliable interpretations of past changes.

## The benefits of synergy: combining sed-eDNA and resurrection ecology

It is apparent from the summaries above of recent work, and other reviews, that these two new fields complement each other, and can greatly enhance traditional palaeoecological information derived from traditional approaches. However, to reach their full potential, knowledge gaps and methodological concerns must be addressed. In this final section we briefly highlight the exciting potential of merging and expanding the analyses of genetic archives to fill the missing ecological links indicated in Fig. 1. By integrating investigations on multiple trophic levels, and combining them with sed-eDNA analyses, the field of resurrection ecology can be taken a step further toward reconstructing whole-ecosystem responses to environmental change. So far, most experiments on eco-evolutionary dynamics spanning different trophic levels have used simplified systems such as prey-predator dynamics in well-controlled experiments^89^. The methods presented here have the potential to extend this approach to the greater complexity of natural systems. In addition to the examples provided in this paper, here we briefly present examples of such interactions, where temporal trajectories and species interactions, based on existing knowledge, could be reconstructed from sediment sequences.

Most eDNA studies lack even an indirect link to phenotype and therefore cannot connect genetic to phenotypic changes, although functional gene analysis provides this opportunity in some cases. In addition, there may be uncertainty associated with the provenance of the extracted sequences. This problem is especially true for non-model organisms for which many functional pathways are unknown, or gene annotation is based on phylogenetically distant taxa. Furthermore, while palaeogenomics can provide evidence for community changes over time, there is rarely sufficient resolution for population-level analysis (although this might change, see a recent perspective paper^90^), and it cannot provide a direct link to specific changes at the phenotype level and the organisms’ fitness. Resurrection studies can address these limitations.

One of the benefits of sed-eDNA comes from its ability to provide information about organisms that may not leave an obvious fossil morphological record (Figs. 1 and 3). However, as with the development of other biochemical proxies in palaeoecology, for example, pigments and stable isotopes, without a critical context, sed-eDNA results can simply be another stratigraphic profile to be interpreted subjectively. A more sophisticated approach is to use the molecular results to answer questions that are difficult to address with more traditional palaeoecological approaches. Increased nutrient loading to aquatic ecosystems results in greater production and changed species composition and community structure^91^. However, high temporal resolution stratigraphic sequences from eutrophic lakes show considerable temporal variability in diatom species and cyanobacteria. Using a multi-proxy approach with relevant statistical methods, it is clear that increasing nutrient load, grazing by zooplankton or climate forcing cannot explain all of this temporal variability in algal abundance. In this context, sed-eDNA can provide supplementary evidence of other trophic links. For example, the role of chytrid infections in controlling phytoplankton abundance has been underestimated in freshwaters (see review by Frenken et al.^92^). From a longer temporal perspective, combining fossil *Asterionella* abundance with the sed-eDNA record of the chytrid *Zygorhizidium planktocnium* could provide evidence of an important mechanism controlling *Asterionella* abundance at decadal time scales. See also the work of Kyle^85^ discussed above. A combination of resurrection ecology and sed-eDNA could also be used to test the effect of environmental change on biodiversity, e.g. how genetic diversity changes when one member disappears, and to identify possible corresponding phenotypic responses in the remaining species.

## Conclusions: tracing evolution of planktonic food-webs in sediment archives

The science of palaeoecology allows us to reconstruct past changes in biological communities, but has limitations in species coverage and in the type of information that can be inferred about past processes. New developments in resurrection ecology and sedimentary timeseries of eDNA promise to address these gaps and can become powerful tools for predicting futures changes to ecosystem function. Building on our knowledge about preservation and degradation of organic molecules in aquatic sediments, we can develop and optimise sampling and interpretation of these unique historical and ancient continuous time series of genetic and phenotypic adaptation. DNA preserved in dated sediment cores has the potential to increase taxonomic coverage to include key organisms and processes that are missing in the microfossil record. Resurrection ecology further adds the dimension of linking population genetic changes with adaptive trait shifts, and linking both to environmental drivers. Moreover, expanding coverage in terms of species, organism groups and strain numbers will allow us to reconstruct rates and magnitudes of change under both natural and anthropogenic forcing.

We are confident that in a few years we will be able to address, for example, questions about the sources of adaptive variation and their underlying genomic architecture. Using undisturbed aquatic sediment cores is the only way to obtain such continuous records for high-resolution temporal reconstructions. The quantitative data acquired from this approach thus have the potential to illuminate generic adaptive processes and responses in the face of multiple environmental stressors.
